# Crystal structure and Hirshfeld surface analysis of ethyl 4-hy­droxy-1-methyl-5-oxo-2-(thio­phen-2-yl)-2,5-di­hydro-1*H*-pyrrole-3-carboxyl­ate

**DOI:** 10.1107/S205698902601039X

**Published:** 2026-10-23

**Authors:** Nor Habibah Mohd Rosli, Amalina Mohd Tajuddin, Muhamad Azwan Hamali, Siti Syaida Sirat, Mohd Fazli Mohammat

**Affiliations:** a Faculty of Applied Sciences Universiti Teknologi MARA Cawangan Pahang Kampus Jengka 26400 Bandar Pusat Jengka Pahang Malaysia; b Atta-ur-Rahman Institute for Natural Product Discovery (AuRIns), Universiti Teknologi MARA, Kampus Puncak Alam, 42300, Bandar Puncak Alam, Selangor, Malaysia; c Faculty of Applied Sciences, Universiti Teknologi MARA, 40450, Shah Alam, Selangor, Malaysia; d Organic Synthesis Research Laboratory, Institute of Science, Universiti Teknologi MARA, 42300, Bandar Puncak Alam, Selangor, Malaysia; Texas A & M University, USA

**Keywords:** crystal structure, pyrrole, thio­phen­yl, multicom­ponent reactions, Hirshfeld surface analysis

## Abstract

The mol­ecular and crystal structure of the title com­pound, C_12_H_13_NO_4_S, has been determined *via* single-crystal X-ray diffraction. As the com­pound crystallizes in the centrosymmetric space group *P*2_1_/*c*, the crystal is racemic and contains equal numbers of *R* and *S* enanti­omers related by inversion symmetry. The thio­phene substituent is positionally disordered over two orientations arising from rotation about the C4—C5 bond, with refined site occupancies of 0.929 (2) and 0.071 (2) for the major and minor com­ponents, respectively. The major thio­phene ring is essentially planar and adopts a nearly orthogonal orientation with respect to the conjugated enaminone fragment. In the crystal packing, the title mol­ecules are linked by inter­molecular O—H⋯O hy­dro­gen bonds to form one-dimensional chains, which are further supported by weak C—H⋯O inter­actions, giving rise to a three-dimensional supra­molecular architecture. Hirshfeld surface analysis qu­anti­tatively revealed that the crystal packing is predominantly governed by H⋯H (41.5%) and H⋯O/O⋯H (31.0%) contacts, with smaller contributions from other inter­molecular inter­actions.

## Abstract 2

The title ester crystallizes with equal numbers of *R* and *S* enanti­omers related by in­ver­sion symmetry. Mol­ecules are linked by inter­molecular O—H⋯O hy­dro­gen bonds to form one-dimensional chains, which are further supported by weak C—H⋯O inter­actions, giving rise to a three-dimensional supra­molecular architecture.

## 1. Chemical context

Pyrrole derivatives are extensively used as building blocks in pharmaceutical design due to their versatile functionalization and ability to inter­act with a diverse range of biological targets, as well as their anti­microbial, anti­cancer, anti-inflammatory and anti­diabetic activities (Amina & Redouane, 2025 ▸; Yadavalli *et al.*, 2024 ▸). The biological activities of thio­phene-fused pyrroles are increased due to the presence of both heterocyclic systems (Rajender Kumar *et al.*, 2004 ▸; Prashanth *et al.*, 2025 ▸). Some derivatives are fluorescent and have been used as biomarkers for disease diagnosis (Moiseeva *et al.*, 2024 ▸; Ferreira *et al.*, 2020 ▸). Several key synthetic strategies for the synthesis of pyrroles and thio­phene-fused pyrroles have been reported, including multicom­ponent reactions (MCR) (Inglebert *et al.*, 2013 ▸), multi-step reductive aminations (Guguloth & Gubbiyappa, 2024 ▸) and modified Clauson–Kaas pyrrole ring closures on thio­phene templates (Rochais *et al.*, 2004 ▸), to advanced macrocyclic *ortho*-phenyl­ene–pyrrole–thio­phene icosa­mers (Matsuo *et al.*, 2025 ▸).

However, there is limited empirical structural data on thio­phene-substituted pyrrole derivatives although the synthesis of pyrroles is well documented. A definitive description of the mol­ecular geometry and the supra­molecular architecture of ethyl 4-hy­droxy-1-methyl-5-oxo-2-(thio­phen-2-yl)-2,5-di­hydro-1*H*-pyrrole-3-carboxyl­ate is absent in the structural chemistry literature. Thus, the objective of this study is to determine the three-dimensional crystal structure of the title com­pound using single-crystal X-ray diffraction and to analyze the percentage contributions of diverse inter­molecular inter­actions to the crystal packing using Hirshfeld surface analysis.

## 2. Structural commentary

**Figure 1:**
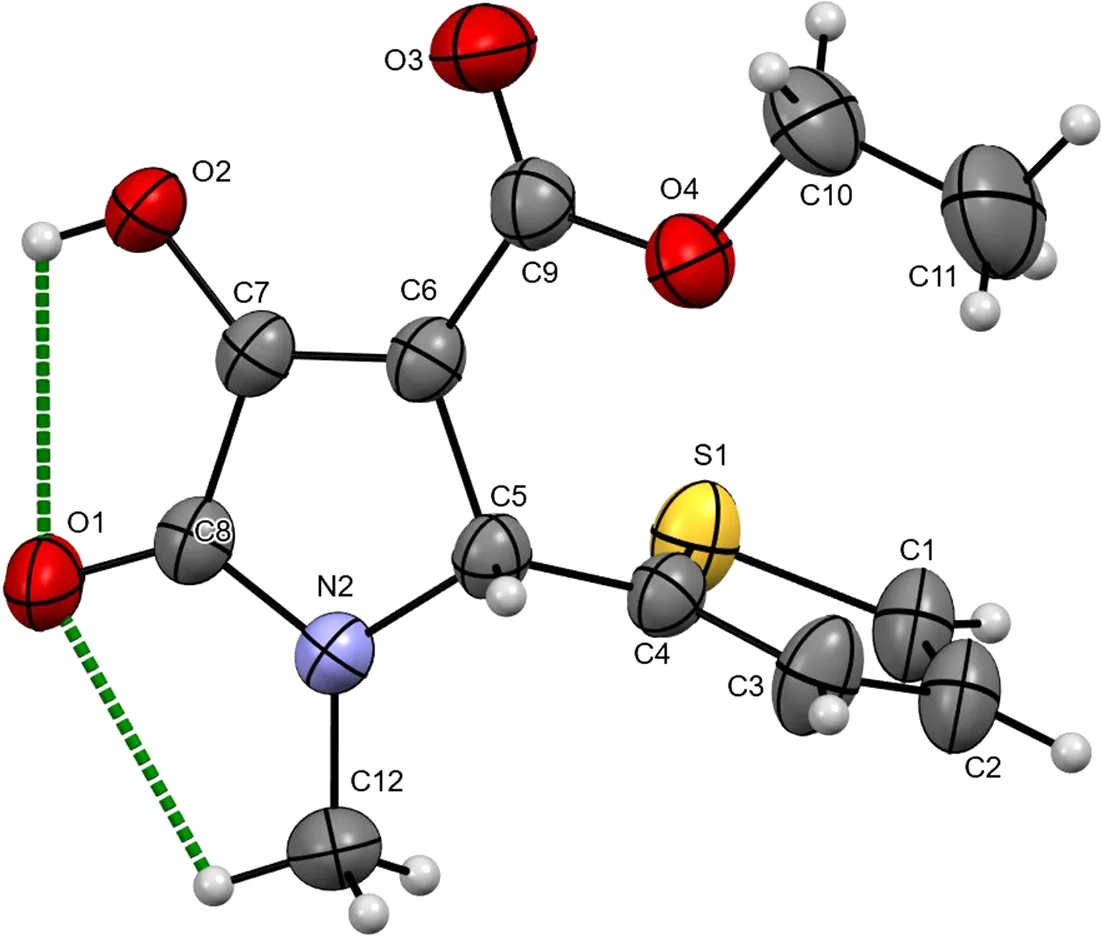
The mol­ecular structure of the title com­pound, showing 50% probability displacement ellipsoids. The minor com­ponent of the disordered thio­phene ring has been omitted for clarity. The O—H⋯O and C—H⋯O intra­molecular contacts are shown as green dashed lines.

The mol­ecular structure of the title com­pound, showing the atom-labelling scheme, is pre­sent­ed in Fig. 1 ▸. The asymmetric unit com­prises one crystallographically independent mol­ecule. The mol­ecule possesses a single stereogenic centre at atom C5. As the structure crystallizes in the centosymmetric space group *P*2_1_/*c*, the crystal is racemic and contains equal numbers of the *R* and *S* enanti­omers related by crystallographic inversion symmetry.
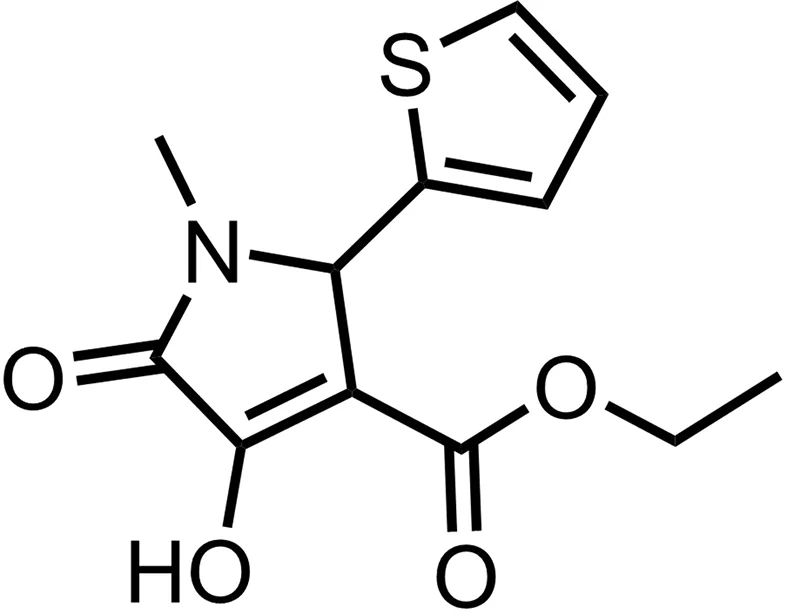


The thio­phene substituent is positionally disordered over two orientations arising from rotation about the C4—C5 bond. The two disorder com­ponents were refined with site-occupancy factors of 0.929 (2) and 0.071 (2), respectively. For the major com­ponent, the thio­phene ring (S1/C1–C4) is essentially planar, with a maximum deviation of 0.006 (1) Å for the C4 atom from the least-squares plane and it is oriented nearly perpendicular to the conjugated enaminone fragment (N2/C5–C8), with a dihedral angle of 89.94 (12)° between the planes, adopting a nearly orthogonal arrangement. The conjugated enaminone fragment is essentially planar, as indicated by the torsion angles C5—N2—C8—C7 = 1.5 (2)°, N2—C8—C7—C6 = 0.2 (2)° and C5—C6—C7—C8 = −1.7 (2)°. A weak intra­molecular O2—H2⋯O1 contact is also present, generating an *S*(5) ring motif and contributing to the observed mol­ecular conformation. In contrast, the tetra­hedral stereogenic atom of C5 introduces a slight distortion into the mol­ecular framework, as reflected by the torsion angles N2—C5—C4—S1 = 61.5 (2)° and C6—C5—C7—C8 = −55.9 (2)°. The carbonyl and ester bond lengths of C8—O1 = 1.229 (2) Å, C9—O3 = 1.201 (3) Å and C9—O4 = 1.323 (3) Å are within the expected range.

## 3. Supra­molecular features

**Figure 2:**
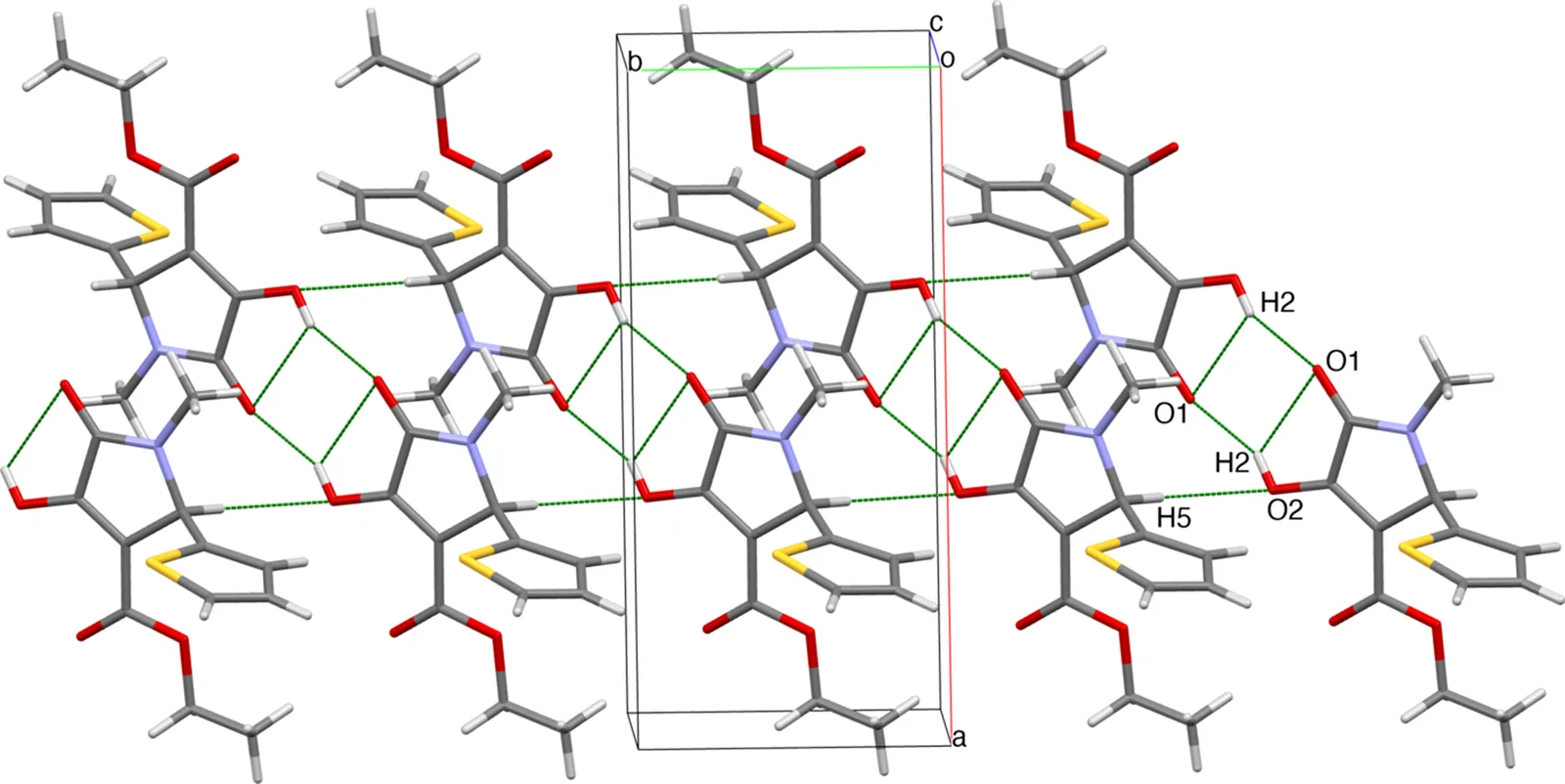
The crystal packing of the title com­pound, viewed down the *c* axis, illustrating the inter­molecular O—H⋯O and C—H⋯O hy­dro­gen-bonding inter­actions, shown as green lines.

In the crystal packing, the mol­ecules are linked through inter­molecular O2—H2⋯O1 hy­dro­gen bonds (Table 1 ▸), generating a one-dimensional hy­dro­gen-bonded chain along the *b* axis. These chains are further stabilized by weak C5—H5⋯O2^ii^ inter­actions, forming the three-dimensional supra­molecular architecture, as shown in Figs. 2 ▸ and 3 ▸.

## 4. Hirshfield surface analysis

**Figure 4:**
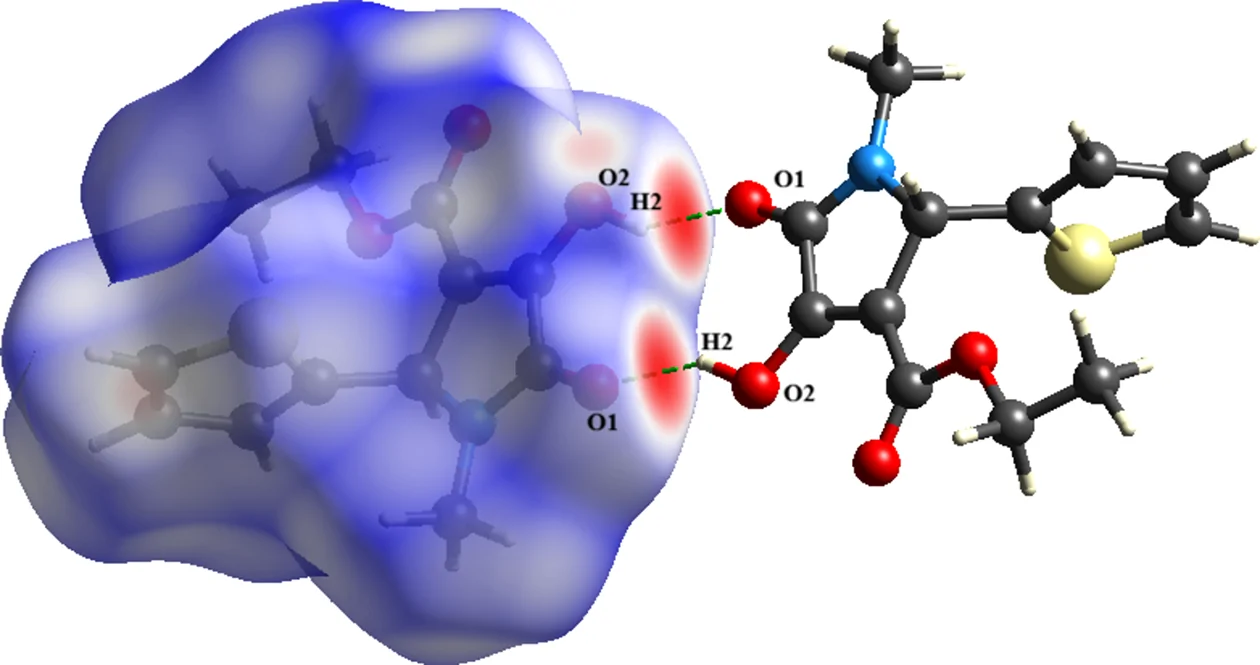
Hirshfeld surface of the title com­pound, mapped over *d*_norm_.

A Hirshfeld surface analysis was performed using *CrystalExplorer21* to examine and qu­antify the inter­molecular contacts governing the crystal packing (Spackman *et al.*, 2021 ▸). Fig. 4 ▸ shows the Hirshfeld surface mapped with the normalized contact distance (*d*_norm_) and reveals two intense red spots. On the *d*_norm_ surface, red, white and blue regions correspond to contacts shorter than, approximately equal to and longer than the sum of the van der Waals radii, respectively. Here, the two intense red spots result from the O2—H2⋯O1 hy­dro­gen bonds (Table 1 ▸).

**Figure 5:**
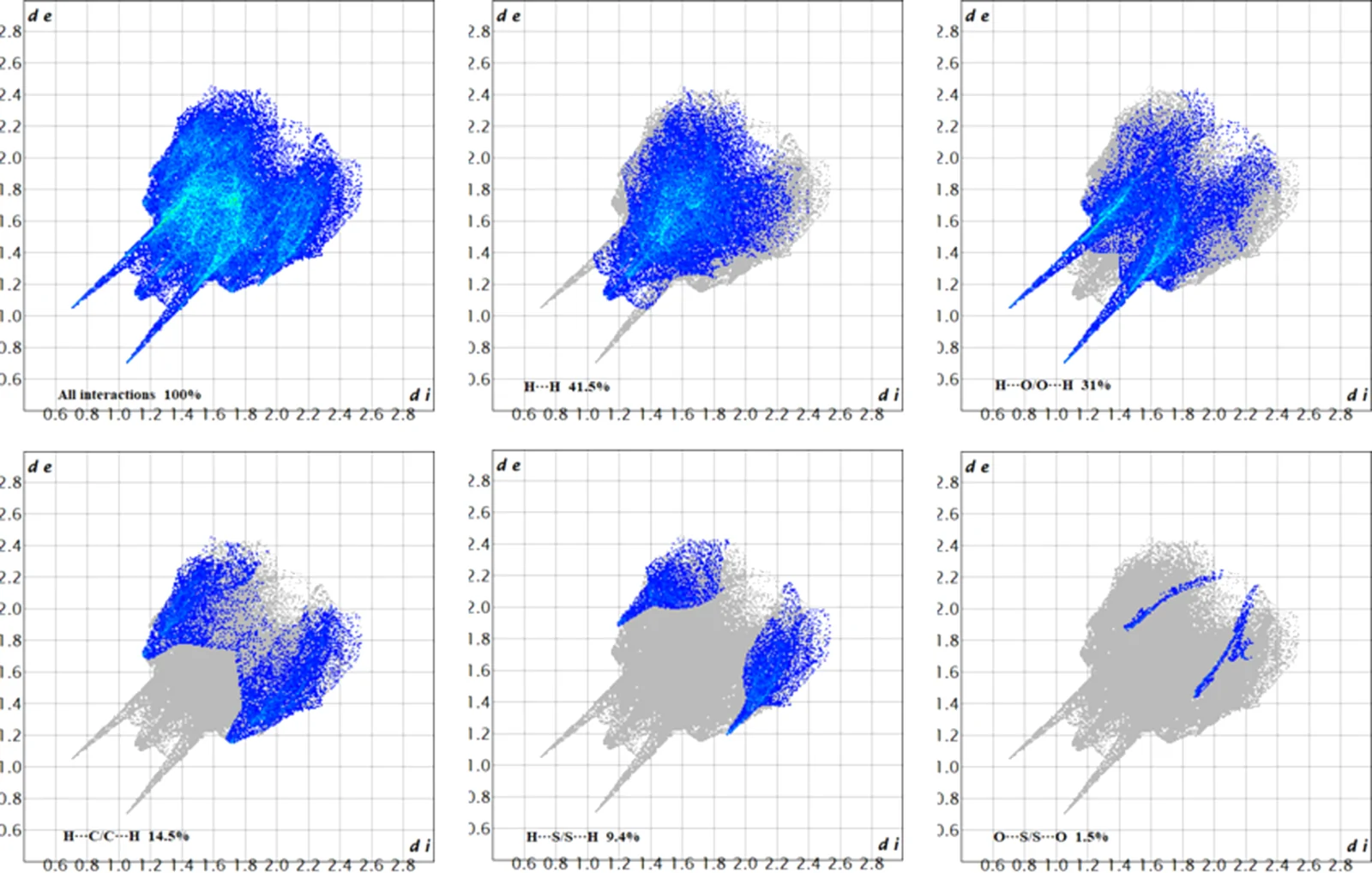
Com­parison of the overall and prominent decom­posed fingerprint plots (>1%) delineated into H⋯H, H⋯O/O⋯H, H⋯C/C⋯H, H⋯S/S⋯H and O⋯S/ S⋯O of the title com­pound.

The percentage contributions of the inter­molecular inter­actions to the total Hirshfeld surface were com­puted by two-dimensional fingerprint plots. As illustrated in Fig. 5 ▸, the *d_i_* versus *d_e_* plots show that H⋯H contacts make the largest contribution (41.5%). The H⋯O/O⋯H contacts contribute 31% to the total Hirshfeld surface and are characterized by a pair of prominent spikes in the two-dimensional fingerprint plot. These features are consistent with the O2—H2⋯O1 hy­dro­gen bonding inter­action observed in the crystal (Table 1 ▸). Other contacts contribute to a lesser extent, namely, H⋯C/C⋯H (14.5%), H⋯S/S⋯H (9.4%) and O⋯S/S⋯O (1.5%).

## 5. Database survey

A search of the Cambridge Structural Database (webCSD accessed August 2026; Groom *et al.*, 2016 ▸) for the title com­pound returned no relevant hits. However, a search with generalized pyrrole derivatives returned a number of hits, including CSD refcodes OHEJIY (Abdul Rashid *et al.*, 2024 ▸), DILYIK (Abdul Rashid *et al.*, 2023 ▸), BOQXEN (del Corte *et al.*, 2019 ▸), EPEVED (Ahankar *et al.*, 2021 ▸), LIFBEJ (Sun *et al.*, 2011 ▸) and MEFYEF (Mal & Mukhopadhyay, 2022 ▸). The structure also is consistent with thio­phene-substituted pyrrole derivatives, namely, DINBUA (Fedoseev *et al.*, 2018 ▸) and GITDEV (Saha & Das, 2017 ▸).

## 6. Synthesis and crystallization

The title com­pound was synthesized using our in-house method described by Mohammat *et al.* (2015 ▸) *via* a multiple-com­ponent reaction of methyl­amine, thio­phene and sodium diethyl oxalacetate. A single yellow crystal of the com­pound, suitable for X-ray diffraction analysis, was grown by slow evaporation at room tem­per­a­ture from ethyl acetate/hexane (yield 0.44 g, 43%). IR (ATR): 1733 (C=O), 1663 (C=O) cm^−1^; ^1^H NMR (400 MHz, chloro­form-*d*): δ 7.29 (*dd*, *J* = 5.0, 1.5 Hz, 1H, HC=C), 7.12–7.06 (*m*, 1H, HC=C), 6.98 (*dd*, *J* = 5.2, 3.5 Hz, 1H, HC=C), 5.33 (*s*, 1H, NCH), 4.27–4.08 (*m*, 2H, CH_2_), 2.88 (*s*, 3H, NCH_3_), 1.16 (*t*, *J* = 7.1 Hz, 3H, CH_3_); ^13^C NMR (101 MHz, chloro­form-*d*): δ 165.19 (C=O), 163.16 (C=O), 157.99 (quat. C), 138.34 (HC=C), 127.96 (HC=C), 126.94 (HC=C), 126.34 (HC=C), 112.35 (C=C), 61.21 (CH_2_), 57.91 (NCH), 27.65 (NCH_3_), 14.06 (CH_3_).

## 7. Refinement

Crystal data, data collection and structure refinement details are summarized in Table 2 ▸. Carbon-bound H atoms were positioned geometrically and refined using a riding model, with C—H distances of 0.93 (aromatic), 0.97 (methyl­ene), 0.98 (methine) and 0.96 Å (meth­yl). The methyl H atoms were refined as rotating groups. Isotropic displacement parameters were constrained to *U*_iso_(H) = 1.2*U*_eq_(C) for aromatic, methyl­ene and methine H atoms, and to 1.5*U*_eq_(C) for methyl H atoms. The hydroxyl H atom was located in a difference Fourier map and refined independently with an isotropic displacement parameter.

## Supplementary Material

Crystal structure: contains datablock(s) I. DOI: 10.1107/S205698902601039X/jy2077sup1.cif

Structure factors: contains datablock(s) I. DOI: 10.1107/S205698902601039X/jy2077Isup3.hkl

Supporting information file. DOI: 10.1107/S205698902601039X/jy2077Isup3.cml

CCDC reference: 2583324

Additional supporting information:  crystallographic information; 3D view; checkCIF report

## Figures and Tables

**Figure 3:**
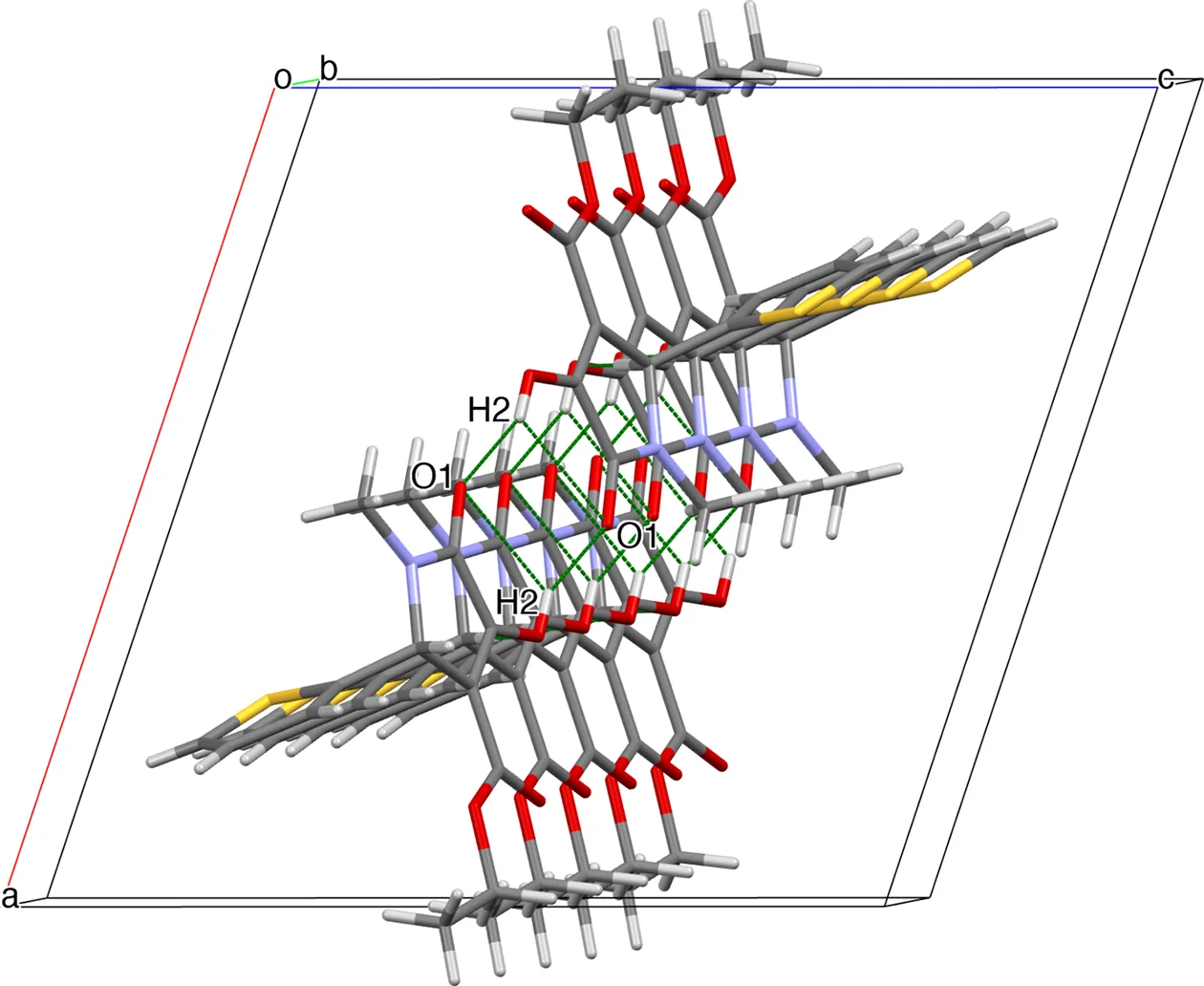
The crystal packing of the title com­pound, viewed down the *b* axis, illustrating the inter­molecular O—H⋯O and C—H⋯O hy­dro­gen-bonding inter­actions, shown as green lines.

**Table 1:** Hydrogen-bond geometry (Å, °)

*D*—H⋯*A*	*D*—H	H⋯*A*	*D*⋯*A*	*D*—H⋯*A*
O2—H2⋯O1	0.77 (3)	2.54 (3)	2.905 (2)	112 (3)
C12—H12*A*⋯O1	0.96	2.55	2.910 (3)	103
C12—H12*A*⋯O1^i^	0.77 (3)	1.94 (3)	2.637 (2)	151 (3)
C5—H5⋯O2^ii^	0.98	2.53	3.489 (2)	166

Symmetry codes: (i) 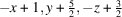; (ii) 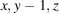.

**Table 2:** Experimental details

Crystal data	
Chemical formula	C_12_H_13_NO_4_S
*M*_r_	267.29
Crystal system, space group	Monoclinic, *P*2_1_/*c*
Temperature (K)	298
*a*, *b*, *c* (Å)	14.3260 (4), 6.3501 (2), 14.7341 (4)
β (°)	108.740 (2)
*V* (Å^3^)	1269.32 (7)
*Z*	4
Radiation type	Mo *K*α
μ (mm^−1^)	0.26
Crystal size (mm)	0.5 × 0.15 × 0.07

Data collection	
Diffractometer	Bruker SMART APEX CCD area detector
No. of measured, independent and observed [*I* > 2σ(*I*)] reflections	23386, 3160, 2243
*R*_int_	0.058
(sin θ/λ)_max_ (Å^−1^)	0.668

Refinement	
*R*[*F*^2^ > 2σ(*F*^2^)], *wR*(*F*^2^), *S*	0.043, 0.118, 1.02
No. of reflections	3160
No. of parameters	176
No. of restraints	12
H-atom treatment	H atoms treated by a mixture of independent and constrained refinement
Δρ_max_, Δρ_min_ (e Å^−3^)	0.27, −0.20

Computer programs: *APEX2* (Bruker, 2014 ▸), *SAINT* (Bruker, 2014 ▸), *SHELXTL* (Sheldrick, 2015*a* ▸), *SHELXL2014* (Sheldrick, 2015*b* ▸), and *OLEX2* (Dolomanov *et al.*, 2009 ▸).

## supplementary crystallographic information

### Ethyl 4-hydroxy-1-methyl-5-oxo-2-(thiophen-2-yl)-2,5-dihydro-1H-pyrrole-3-carboxylate Crystal data

**Table:**

C_12_H_13_NO_4_S	*F*(000) = 560
*M**_r_* = 267.29	*D*_x_ = 1.399 Mg m^−^^3^
Monoclinic, *P*2_1_/*c*	Mo *K*α radiation, λ = 0.71073 Å
*a* = 14.3260 (4) Å	Cell parameters from 5165 reflections
*b* = 6.3501 (2) Å	θ = 2.8–24.0°
*c* = 14.7341 (4) Å	µ = 0.26 mm^−^^1^
β = 108.740 (2)°	*T* = 298 K
*V* = 1269.32 (7) Å^3^	Plate, colourless
*Z* = 4	0.5 × 0.15 × 0.07 mm

### Ethyl 4-hydroxy-1-methyl-5-oxo-2-(thiophen-2-yl)-2,5-dihydro-1H-pyrrole-3-carboxylate Data collection

**Table:**

Bruker SMART APEX CCD area detector diffractometer	2243 reflections with *I* > 2σ(*I*)
Radiation source: sealed tube	*R*_int_ = 0.058
Graphite monochromator	θ_max_ = 28.4°, θ_min_ = 3.5°
Detector resolution: 8 pixels mm^-1^	*h* = −19→19
ω and φ scans	*k* = −8→8
23386 measured reflections	*l* = −18→19
3160 independent reflections	

### Ethyl 4-hydroxy-1-methyl-5-oxo-2-(thiophen-2-yl)-2,5-dihydro-1H-pyrrole-3-carboxylate Refinement

**Table:**

Refinement on *F*^2^	Primary atom site location: dual
Least-squares matrix: full	Hydrogen site location: mixed
*R*[*F*^2^ > 2σ(*F*^2^)] = 0.043	H atoms treated by a mixture of independent and constrained refinement
*wR*(*F*^2^) = 0.118	*w* = 1/[σ^2^(*F*_o_^2^) + (0.0497*P*)^2^ + 0.4448*P*] where *P* = (*F*_o_^2^ + 2*F*_c_^2^)/3
*S* = 1.02	(Δ/σ)_max_ < 0.001
3160 reflections	Δρ_max_ = 0.27 e Å^−^^3^
176 parameters	Δρ_min_ = −0.20 e Å^−^^3^
12 restraints	

### Ethyl 4-hydroxy-1-methyl-5-oxo-2-(thiophen-2-yl)-2,5-dihydro-1H-pyrrole-3-carboxylate Special details

**Table:**

Geometry. All esds (except the esd in the dihedral angle between two l.s. planes) are estimated using the full covariance matrix. The cell esds are taken into account individually in the estimation of esds in distances, angles and torsion angles; correlations between esds in cell parameters are only used when they are defined by crystal symmetry. An approximate (isotropic) treatment of cell esds is used for estimating esds involving l.s. planes.The equation of the plane is of the form: P * x + Q * y + R * z - S = 0 where P, Q, R, S are constants and x, y, z are fractional coordinates. P = 10.577 (11), Q = 1.349 (6), R = 5.437 (13), S = 10.079 (3) Atom Distance x y z * S1: -0.005 (1) 0.7316 0.5380 0.2962 * C1: 0.003 (2) 0.7824 0.3487 0.2457 * C2: 0.001 (3) 0.7849 0.1612 0.2870 * C3: -0.005 (3) 0.7442 0.1626 0.3647 * C4: 0.006 (2) 0.7131 0.3639 0.3773 * P = -7.400 (12), Q = -2.756 (6), R = 12.745 (10), S = -0.384 (10) Atom Distance x y z * N2: -0.012 (2) 0.5654 0.5073 0.4069 * C5: 0.014 (2) 0.6665 0.4292 0.4508 * C6: -0.013 (2) 0.7152 0.6096 0.5160 * C7: 0.006 (2) 0.6481 0.7589 0.5107 * C8: 0.004 (2) 0.5518 0.6964 0.4411 *

### Ethyl 4-hydroxy-1-methyl-5-oxo-2-(thiophen-2-yl)-2,5-dihydro-1H-pyrrole-3-carboxylate Fractional atomic coordinates and isotropic or equivalent isotropic displacement parameters (Å^2^)

**Table:**

	*x*	*y*	*z*	*U*_iso_*/*U*_eq_	Occ. (<1)
S1	0.73146 (5)	0.53827 (9)	0.29597 (5)	0.0537 (2)	0.929 (2)
O1	0.47515 (9)	0.7992 (2)	0.41956 (10)	0.0537 (4)	
O2	0.65899 (10)	0.9413 (2)	0.55704 (10)	0.0469 (3)	
O3	0.85752 (11)	0.7521 (3)	0.62993 (13)	0.0744 (5)	
O4	0.86123 (10)	0.4271 (2)	0.57403 (10)	0.0551 (4)	
N2	0.56557 (10)	0.5076 (2)	0.40703 (11)	0.0414 (4)	
C11	1.0011 (2)	0.2150 (5)	0.5940 (3)	0.0957 (9)	
H11A	0.993122	0.232038	0.527155	0.144*	
H11B	0.964010	0.094891	0.602372	0.144*	
H11C	1.069547	0.194408	0.629167	0.144*	
C10	0.96487 (16)	0.4060 (4)	0.62976 (18)	0.0692 (6)	
H10A	0.972688	0.391347	0.697333	0.083*	
H10B	1.001395	0.529001	0.621344	0.083*	
C9	0.81827 (13)	0.6095 (3)	0.57917 (13)	0.0446 (4)	
C6	0.71514 (12)	0.6098 (3)	0.51600 (12)	0.0381 (4)	
C5	0.66662 (12)	0.4295 (3)	0.45074 (12)	0.0381 (4)	
H5	0.664628	0.307015	0.490526	0.046*	
C4	0.71304 (12)	0.3640 (3)	0.37738 (12)	0.0383 (4)	
C1	0.7825 (2)	0.3461 (4)	0.24489 (18)	0.0537 (5)	0.929 (2)
H1	0.806754	0.369330	0.194303	0.064*	0.929 (2)
C2	0.78308 (18)	0.1595 (4)	0.28646 (17)	0.0537 (5)	0.929 (2)
H2A	0.806150	0.035786	0.267398	0.064*	0.929 (2)
C3	0.7444 (4)	0.1732 (5)	0.3630 (3)	0.0574 (8)	0.929 (2)
H3	0.740980	0.057679	0.400569	0.069*	0.929 (2)
C8	0.55184 (13)	0.6964 (3)	0.44110 (12)	0.0396 (4)	
C7	0.64828 (13)	0.7589 (3)	0.51072 (12)	0.0374 (4)	
C12	0.49014 (14)	0.3923 (3)	0.33459 (15)	0.0537 (5)	
H12A	0.427934	0.462546	0.322046	0.081*	
H12B	0.485397	0.252043	0.357042	0.081*	
H12C	0.507151	0.385933	0.276719	0.081*	
H2	0.606 (2)	0.996 (4)	0.5500 (17)	0.072 (8)*	
C3A	0.761 (3)	0.499 (3)	0.337 (2)	0.0537 (2)	0.071 (2)
H3A	0.777749	0.637631	0.355350	0.064*	0.071 (2)
C2A	0.782 (3)	0.393 (4)	0.260 (2)	0.0537 (5)	0.071 (2)
H2AA	0.775026	0.442555	0.199384	0.064*	0.071 (2)
C1A	0.814 (2)	0.195 (4)	0.299 (2)	0.0537 (5)	0.071 (2)
H1A	0.864381	0.118193	0.288507	0.064*	0.071 (2)
S1A	0.7427 (17)	0.1129 (18)	0.3721 (15)	0.0574 (8)	0.071 (2)

### Ethyl 4-hydroxy-1-methyl-5-oxo-2-(thiophen-2-yl)-2,5-dihydro-1H-pyrrole-3-carboxylate Atomic displacement parameters (Å^2^)

**Table:**

	*U*^11^	*U*^22^	*U*^33^	*U*^12^	*U*^13^	*U*^23^
S1	0.0706 (4)	0.0410 (3)	0.0575 (4)	0.0124 (2)	0.0316 (3)	0.0121 (2)
O1	0.0443 (7)	0.0526 (8)	0.0589 (8)	0.0142 (6)	0.0091 (6)	−0.0119 (6)
O2	0.0451 (7)	0.0371 (7)	0.0558 (8)	0.0030 (6)	0.0125 (6)	−0.0123 (6)
O3	0.0545 (9)	0.0623 (10)	0.0891 (12)	0.0031 (7)	−0.0009 (8)	−0.0281 (9)
O4	0.0428 (7)	0.0519 (8)	0.0609 (8)	0.0122 (6)	0.0030 (6)	−0.0044 (6)
N2	0.0385 (8)	0.0362 (8)	0.0487 (8)	0.0009 (6)	0.0127 (6)	−0.0072 (6)
C11	0.0624 (15)	0.0839 (19)	0.133 (3)	0.0273 (14)	0.0205 (16)	0.0003 (18)
C10	0.0446 (11)	0.0753 (16)	0.0756 (15)	0.0139 (11)	0.0025 (10)	0.0026 (12)
C9	0.0442 (10)	0.0444 (10)	0.0446 (10)	0.0044 (8)	0.0133 (8)	−0.0001 (8)
C6	0.0432 (9)	0.0339 (8)	0.0379 (8)	0.0038 (7)	0.0139 (7)	0.0012 (7)
C5	0.0412 (9)	0.0307 (8)	0.0435 (9)	0.0040 (7)	0.0151 (7)	0.0018 (7)
C4	0.0409 (9)	0.0331 (9)	0.0408 (9)	0.0042 (7)	0.0129 (7)	0.0007 (7)
C1	0.0596 (9)	0.0557 (11)	0.0507 (10)	0.0180 (9)	0.0245 (8)	0.0016 (8)
C2	0.0596 (9)	0.0557 (11)	0.0507 (10)	0.0180 (9)	0.0245 (8)	0.0016 (8)
C3	0.0816 (14)	0.0372 (19)	0.0626 (16)	0.014 (2)	0.0359 (11)	0.0087 (18)
C8	0.0423 (9)	0.0367 (9)	0.0406 (9)	0.0039 (7)	0.0147 (7)	−0.0006 (7)
C7	0.0447 (9)	0.0320 (8)	0.0375 (8)	0.0015 (7)	0.0161 (7)	−0.0005 (7)
C12	0.0459 (10)	0.0533 (12)	0.0592 (12)	−0.0058 (9)	0.0133 (9)	−0.0175 (9)
C3A	0.0706 (4)	0.0410 (3)	0.0575 (4)	0.0124 (2)	0.0316 (3)	0.0121 (2)
C2A	0.0596 (9)	0.0557 (11)	0.0507 (10)	0.0180 (9)	0.0245 (8)	0.0016 (8)
C1A	0.0596 (9)	0.0557 (11)	0.0507 (10)	0.0180 (9)	0.0245 (8)	0.0016 (8)
S1A	0.0816 (14)	0.0372 (19)	0.0626 (16)	0.014 (2)	0.0359 (11)	0.0087 (18)

### Ethyl 4-hydroxy-1-methyl-5-oxo-2-(thiophen-2-yl)-2,5-dihydro-1H-pyrrole-3-carboxylate Geometric parameters (Å, º)

**Table:**

S1—C4	1.7137 (17)	C5—H5	0.9800
S1—C1	1.716 (2)	C5—C4	1.498 (2)
O1—C8	1.229 (2)	C4—C3	1.333 (4)
O2—C7	1.328 (2)	C4—C3A	1.355 (16)
O2—H2	0.81 (3)	C4—S1A	1.658 (12)
O3—C9	1.194 (2)	C1—H1	0.9300
O4—C10	1.454 (2)	C1—C2	1.333 (3)
O4—C9	1.325 (2)	C2—H2A	0.9300
N2—C5	1.469 (2)	C2—C3	1.410 (4)
N2—C8	1.338 (2)	C3—H3	0.9300
N2—C12	1.450 (2)	C8—C7	1.486 (2)
C11—H11A	0.9600	C12—H12A	0.9600
C11—H11B	0.9600	C12—H12B	0.9600
C11—H11C	0.9600	C12—H12C	0.9600
C11—C10	1.481 (4)	C3A—H3A	0.9300
C10—H10A	0.9700	C3A—C2A	1.420 (18)
C10—H10B	0.9700	C2A—H2AA	0.9300
C9—C6	1.472 (2)	C2A—C1A	1.393 (18)
C6—C5	1.513 (2)	C1A—H1A	0.9300
C6—C7	1.331 (2)	C1A—S1A	1.778 (18)
			
C4—S1—C1	91.76 (11)	C3A—C4—C5	123.4 (10)
C7—O2—H2	110.9 (18)	C3A—C4—S1A	114.4 (11)
C9—O4—C10	117.19 (16)	S1—C1—H1	124.1
C8—N2—C5	112.38 (14)	C2—C1—S1	111.82 (19)
C8—N2—C12	124.32 (15)	C2—C1—H1	124.1
C12—N2—C5	123.29 (14)	C1—C2—H2A	124.3
H11A—C11—H11B	109.5	C1—C2—C3	111.5 (2)
H11A—C11—H11C	109.5	C3—C2—H2A	124.3
H11B—C11—H11C	109.5	C4—C3—C2	115.0 (3)
C10—C11—H11A	109.5	C4—C3—H3	122.5
C10—C11—H11B	109.5	C2—C3—H3	122.5
C10—C11—H11C	109.5	O1—C8—N2	127.31 (17)
O4—C10—C11	106.6 (2)	O1—C8—C7	126.29 (16)
O4—C10—H10A	110.4	N2—C8—C7	106.40 (14)
O4—C10—H10B	110.4	O2—C7—C6	128.92 (17)
C11—C10—H10A	110.4	O2—C7—C8	121.08 (15)
C11—C10—H10B	110.4	C6—C7—C8	110.00 (15)
H10A—C10—H10B	108.6	N2—C12—H12A	109.5
O3—C9—O4	124.40 (17)	N2—C12—H12B	109.5
O3—C9—C6	124.76 (17)	N2—C12—H12C	109.5
O4—C9—C6	110.82 (15)	H12A—C12—H12B	109.5
C9—C6—C5	124.33 (15)	H12A—C12—H12C	109.5
C7—C6—C9	126.52 (16)	H12B—C12—H12C	109.5
C7—C6—C5	109.03 (15)	C4—C3A—H3A	125.9
N2—C5—C6	102.13 (13)	C4—C3A—C2A	108.2 (18)
N2—C5—H5	108.4	C2A—C3A—H3A	125.9
N2—C5—C4	112.38 (14)	C3A—C2A—H2AA	128.4
C6—C5—H5	108.4	C1A—C2A—C3A	103.3 (19)
C4—C5—C6	116.67 (14)	C1A—C2A—H2AA	128.4
C4—C5—H5	108.4	C2A—C1A—H1A	125.2
C5—C4—S1	121.96 (12)	C2A—C1A—S1A	109.5 (18)
C5—C4—S1A	118.7 (6)	S1A—C1A—H1A	125.2
C3—C4—S1	109.9 (2)	C4—S1A—C1A	87.2 (9)
C3—C4—C5	128.2 (2)		
			
S1—C4—C3—C2	−0.5 (5)	C5—N2—C8—O1	−178.65 (17)
S1—C1—C2—C3	−1.8 (4)	C5—N2—C8—C7	1.45 (19)
O1—C8—C7—O2	−0.2 (3)	C5—C6—C7—O2	178.89 (16)
O1—C8—C7—C6	−179.82 (17)	C5—C6—C7—C8	−1.51 (19)
O3—C9—C6—C5	178.93 (19)	C5—C4—C3—C2	178.6 (3)
O3—C9—C6—C7	−5.4 (3)	C5—C4—C3A—C2A	−171 (2)
O4—C9—C6—C5	−2.6 (2)	C5—C4—S1A—C1A	−166.1 (12)
O4—C9—C6—C7	173.00 (16)	C4—S1—C1—C2	1.4 (2)
N2—C5—C4—S1	61.47 (18)	C4—C3A—C2A—C1A	−43 (4)
N2—C5—C4—C3	−117.6 (4)	C1—S1—C4—C5	−179.67 (17)
N2—C5—C4—C3A	86 (2)	C1—S1—C4—C3	−0.4 (3)
N2—C5—C4—S1A	−115.8 (10)	C1—C2—C3—C4	1.6 (5)
N2—C8—C7—O2	179.72 (15)	C8—N2—C5—C6	−2.25 (18)
N2—C8—C7—C6	0.08 (19)	C8—N2—C5—C4	−128.06 (15)
C10—O4—C9—O3	−4.0 (3)	C7—C6—C5—N2	2.22 (18)
C10—O4—C9—C6	177.58 (17)	C7—C6—C5—C4	125.17 (16)
C9—O4—C10—C11	−166.2 (2)	C12—N2—C5—C6	177.25 (16)
C9—C6—C5—N2	178.53 (15)	C12—N2—C5—C4	51.4 (2)
C9—C6—C5—C4	−58.5 (2)	C12—N2—C8—O1	1.9 (3)
C9—C6—C7—O2	2.7 (3)	C12—N2—C8—C7	−178.04 (16)
C9—C6—C7—C8	−177.71 (16)	C3A—C4—S1A—C1A	−6 (2)
C6—C5—C4—S1	−56.0 (2)	C3A—C2A—C1A—S1A	38 (3)
C6—C5—C4—C3	124.9 (4)	C2A—C1A—S1A—C4	−20 (2)
C6—C5—C4—C3A	−31 (2)	S1A—C4—C3A—C2A	30 (3)
C6—C5—C4—S1A	126.8 (10)		

### Ethyl 4-hydroxy-1-methyl-5-oxo-2-(thiophen-2-yl)-2,5-dihydro-1H-pyrrole-3-carboxylate Hydrogen-bond geometry (Å, º)

**Table:**

*D*—H···*A*	*D*—H	H···*A*	*D*···*A*	*D*—H···*A*
O2—H2···O1	0.77 (3)	2.54 (3)	2.905 (2)	112 (3)
C12—H12*A*···O1	0.96	2.55	2.910 (3)	103
C12—H12*A*···O1^i^	0.77 (3)	1.94 (3)	2.637 (2)	151 (3)
C5—H5···O2^ii^	0.98	2.53	3.489 (2)	166

Symmetry codes: (i) −*x*+1, *y*+5/2, −*z*+3/2; (ii) *x*, *y*−1, *z*.

## References

[bb1] Abdul Rashid, F. N. A., Bacho, M. Z., Slawin, A. M. Z., Abdul Manan, M. A. F., Johari, S. A. & Mohammat, M. F. (2023). *Z. Kristallogr. New Cryst. Struct.* **238**, 113–115.

[bb2] Abdul Rashid, F. N. A., Mohammat, M. F., Abdul Manan, M. A. F., Bakhtiar Afendy, A. A. A., Cordes, D. B. & McKay, A. P. (2024). *IUCrData* **9**, x241222. 10.1107/S2414314624012227 PMC11701760 39777006

[bb3] Ahankar, H., Ramazani, A., Saeidian, H., Ślepokura, K. & Lis, T. (2021). *J. Struct. Chem.* **62**, 47–57.

[bb4] Amina, B. & Redouane, B. (2025). *Curr. Top. Med. Chem.* **25**, 461–492. 10.2174/0115680266307696240708115422 39069813

[bb5] Bruker (2014). *APEX2* and *SAINT*. Bruker AXS Inc., Madison, Wisconsin, USA.

[bb7] Corte, X., Martinez de Marigorta, E., Palacios, F. & Vicario, J. (2019). *Molecules* **24**, 2951, 1–11. 10.3390/molecules24162951 PMC6719937 31416281

[bb8] Dolomanov, O. V., Bourhis, L. J., Gildea, R. J., Howard, J. A. K. & Puschmann, H. (2009). *J. Appl. Cryst.* **42**, 339–341.

[bb9] Fedoseev, S. V., Belikov, M. Y., Ievlev, M. Y., Ershov, O. V. & Tafeenko, V. A. (2018). *Res. Chem. Intermed.* **44**, 3565–3579.

[bb10] Ferreira, J. R. M., Nunes da Silva, R., Rocha, J., Silva, A. M. S. & Guieu, S. (2020). *Synlett* **31**, 632–634.

[bb11] Groom, C. R., Bruno, I. J., Lightfoot, M. P. & Ward, S. C. (2016). *Acta Cryst.* B**72**, 171–179. 10.1107/S2052520616003954 PMC4822653 27048719

[bb12] Guguloth, R. & Gubbiyappa, S. K. (2024). *ajc* **36**, 489–497.

[bb13] Inglebert, S. A., Arun, Y., Sethusankar, K. & Perumal, P. T. (2013). *Acta Cryst.* E**69**, o1585. 10.1107/S1600536813025890 PMC3790442 24098261

[bb14] Mal, K. & Mukhopadhyay, C. (2022). *J. Mol. Struct.* **1265**, 133377–133385.

[bb15] Matsuo, Y., Nakagawa, A., Seki, S. & Tanaka, T. (2025). *Beilstein J. Org. Chem.* **21**, 1561–1567. 10.3762/bjoc.21.119 PMC12320735 40761760

[bb16] Mohammat, M. F., Mansor, N. S., Shaameri, Z. & Hamzah, A. S. (2015). *J. Korean Chem. Soc.* **59**, 31–35.

[bb17] Moiseeva, E. O., Gorbunova, E. A. & Dubinina, T. V. (2024). *Biochemistry (Moscow), Supplement Series B: Biomedical Chemistry* **18**, 15–45.

[bb18] Prashanth, G. K., Lalithamba, H. S., Srilatha, R., Rashmi, K. V., Bhagya, N. P. & Gadewar, M. (2025). *Thienopyrroles-Emerging Therapeutic Agents in Modern Medicine: A Review of Medical Applications*, in *Examining Biological Relevance of Fused S-Heterocycles*, pp. 111–134. Hershey, USA: IGI Global Scientific Publishing.

[bb19] Rajender Kumar, P., Raju, S., Satish Goud, P., Sailaja, M., Sarma, M. R., Om Reddy, G., Prem Kumar, M., Reddy, V. V. R. M. K., Suresh, T. & Hegde, P. (2004). *Bioorg. Med. Chem.* **12**, 1221–1230. 10.1016/j.bmc.2003.11.003 14980634

[bb20] Rochais, C., Lescot, E., Lisowski, V., Lepailleur, A., Santos, J. S. O., Bureau, R., Dallemagne, P., Meijer, L. & Rault, S. (2004). *J. Enzyme Inhib. Med. Chem.* **19**, 585–593. 10.1080/14756360400004565 15662962

[bb21] Saha, M. & Das, A. R. (2017). *ChemistrySelect* **2**, 10249–10260.

[bb22] Sheldrick, G. M. (2015*a*). *Acta Cryst.* A**71**, 3–8.

[bb23] Sheldrick, G. M. (2015*b*). *Acta Cryst.* C**71**, 3–8.

[bb24] Spackman, P. R., Turner, M. J., McKinnon, J. J., Wolff, S. K., Grimwood, D. J., Jayatilaka, D. & Spackman, M. A. (2021). *J. Appl. Cryst.* **54**, 1006–1011. 10.1107/S1600576721002910 PMC8202033 34188619

[bb25] Sun, J., Wu, Q., Xia, E.-Y. & Yan, C.-G. (2011). *Eur. J. Org. Chem.* **2011**, 2981–2986.

[bb26] Yadavalli, V. D. N., Kokkirala, S. & Avudoddi, V. (2024). *Recent research advances in the aqueous phase synthesis of pyrroles*, in *Pyrrole: Synthesis and Applications*, pp. 37–52. New York: Nova Science Publishers.

